# Nondestructive In Situ Measurement Method for Kernel Moisture Content in Corn Ear

**DOI:** 10.3390/s16122196

**Published:** 2016-12-20

**Authors:** Han-Lin Zhang, Qin Ma, Li-Feng Fan, Peng-Fei Zhao, Jian-Xu Wang, Xiao-Dong Zhang, De-Hai Zhu, Lan Huang, Dong-Jie Zhao, Zhong-Yi Wang

**Affiliations:** 1 College of Information and Electrical Engineering, China Agricultural University, Beijing 100083, China; zhang-hanlin@cau.edu.cn (H.-L.Z.); sockline@163.com (Q.M.); zgndnd_flfwxj@cau.edu.cn (L.-F.F.); zgndndzpf@cau.edu.cn (P.-F.Z.); wangjianxu@cau.edu.cn (J.-X.W.); zhangxd@cau.edu.cn (X.-D.Z.); zhudehai@cau.edu.cn (D.-H.Z.); hlan@cau.edu.cn (L.H.); zdj_cau@cau.edu.cn (D.-J.Z.); 2 Key Laboratory of Agricultural Information Acquisition Technology (Beijing), Ministry of Agriculture, Beijing 100083, China; 3 Modern Precision Agriculture System Integration Research Key Laboratory, Ministry of Education, Beijing 100083, China

**Keywords:** corn ear, moisture, nondestructive measurement, high-frequency detection, impedance

## Abstract

Moisture content is an important factor in corn breeding and cultivation. A corn breed with low moisture at harvest is beneficial for mechanical operations, reduces drying and storage costs after harvesting and, thus, reduces energy consumption. Nondestructive measurement of kernel moisture in an intact corn ear allows us to select corn varieties with seeds that have high dehydration speeds in the mature period. We designed a sensor using a ring electrode pair for nondestructive measurement of the kernel moisture in a corn ear based on a high-frequency detection circuit. Through experiments using the effective scope of the electrodes’ electric field, we confirmed that the moisture in the corn cob has little effect on corn kernel moisture measurement. Before the sensor was applied in practice, we investigated temperature and conductivity effects on the output impedance. Results showed that the temperature was linearly related to the output impedance (both real and imaginary parts) of the measurement electrodes and the detection circuit’s output voltage. However, the conductivity has a non-monotonic dependence on the output impedance (both real and imaginary parts) of the measurement electrodes and the output voltage of the high-frequency detection circuit. Therefore, we reduced the effect of conductivity on the measurement results through measurement frequency selection. Corn moisture measurement results showed a quadric regression between corn ear moisture and the imaginary part of the output impedance, and there is also a quadric regression between corn kernel moisture and the high-frequency detection circuit output voltage at 100 MHz. In this study, two corn breeds were measured using our sensor and gave *R*^2^ values for the quadric regression equation of 0.7853 and 0.8496.

## 1. Introduction

Corn is one of the world’s most widely used food crops. Global corn yields in 2015–2016 were 1.0111 billion tons [1]. The moisture content of corn is an important factor in corn breeding and cultivation. Corn breeds with low moisture content offer the advantages of simple mechanical operations and reduced drying and storage costs after harvest and, thus, reduce energy consumption. Since Miller et al. [2] discovered that the moisture content is higher in later-maturing varieties of corn at harvest, a great deal of work has been performed on the corn kernel filling periods and the physiological maturity of seeds by both breeders and botanists. Kang and co-researchers [3] found that there is a significant linear correlation between the grain fill rate during the filling period and the grain-moisture reduction rate during the physiological maturity of seeds. A corn breed would have a higher rate of grain-moisture reduction when it has a higher grain fill rate, and then the grain moisture content would be lower at harvest. It is difficult to harvest, dehydrate, store, translate, and process corn breeds that have high moisture content. Corn breeders have been very concerned about the problem of how to screen and breed corn with low moisture content at harvest, especially when the corn breed has a high grain-moisture reduction rate during the physiological maturity of the seeds [4].

The conventional method used for grain moisture measurement is the drying method, but this method is time-consuming and has complex procedures that can cause serious damage to the samples. Most conventional methods cannot meet the requirements for nondestructive detection of corn kernel moisture in corn ears. Many previous studies have attempted to overcome these limitations. In 1908, Briggs et al. [5] found that there was a significant linear correlation between the natural logarithm of wheat’s electronic resistance and its moisture content. Since then, rapid grain moisture measurement methods based on electrical and dielectric property measurements have been developed. Over the last century, numerous researchers have developed different moisture detection methods based on the electrical and dielectric properties of specific varieties of grain [6,7,8,9,10]. For corn ear moisture content measurement, Kang et al. [11] proposed a type of electronic probe instrument that was used to detect the moisture content of the corn ear directly to determine the moisture content of the corn kernels. The instrument is a type of portable resistance meter with two electrodes. We can detect the moisture in corn kernels by acquiring the current value after insertion of the probe into a corn cob through the corn bracts and the grain. Kang et al. [12] subsequently designed another type of electronic probe with needle-type electrodes to detect the moisture content of corn kernels. A similar type of moisture meter was used for corn moisture detection in the field by Freppon et al. [13]. In this instrument, they used an electronic display rather than the traditional pointer instructions. Xiang et al. [14,15] repurposed the MT808 lumber moisture meter (Electrophysics, London, ON, Canada). They compared the corn ear moisture content measured using the MT808 with that from a prediction model and measured data from the oven drying method. When the instrument was used in the field, the corn ear moisture content was detected quickly and conveniently by simply inserting the sensor into the corn ear. Andrei et al. [16] adopted a similar method to that of Xiang et al. [14,15] to realize rapid corn ear moisture measurements. All of these studies could measure the corn ear moisture quickly and conveniently, and contributed to the development of measurement methods for corn ear moisture. However, for selection of a corn breed with a high grain-moisture reduction rate, rapid and nondestructive in situ detection of the moisture content of kernels in a corn ear remains challenging. First, we must measure the grain filling rate and the dehydration rate in vivo during the growing period of the corn. Second, we must obtain the corn kernel moisture in situ immediately after harvesting. We hope that this method will allow us to measure kernel moisture content without waiting for the moisture to be reduced to an appropriate value for threshing. This will enable a production estimate to be made easily and quickly. In our study, we conducted further research into a nondestructive method for measurement of kernel moisture content in corn ears. Our aim is to determine the corn kernel moisture content quickly and conveniently on the condition that no physical damage is caused to the corn ear. Based on the obvious difference between the dielectric constant of the water and that of the dry matter of the corn, we proposed a nondestructive method for in situ measurement of the corn kernel moisture in the corn ear through appropriate electrode design for radio-frequency operation. 

## 2. Materials and Methods 

### 2.1. Measurement Principle

Corn is largely composed of water, starch, proteins, and fats. The water and the starch are the main components. The dielectric constant of the water is well known to be 76.47 when measured at a frequency of 100 MHz at 30 °C [17], and the dielectric constants of the starch, proteins, and other substances range from approximately 1 to 8 [18,19]. Corn’s hybrid dielectric constant is largely influenced by its moisture content, and the dielectric constant affects the measurement electrode’s impedance. Therefore, changes in the electrode impedance could indirectly reflect the corn’s moisture content.

**Figure 1:**
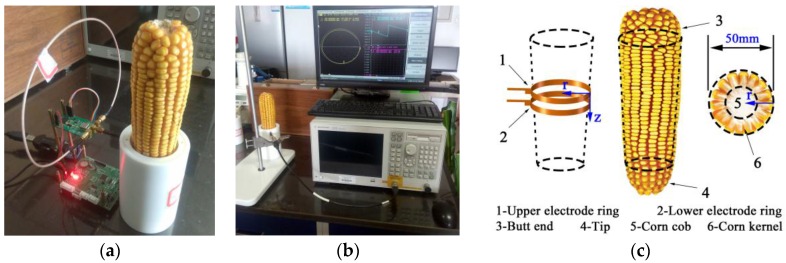
Experimental setup for in situ measurement of corn kernels in a corn ear. (**a**) The device prototype with the sensor; a high-frequency detection circuit is connected to the measurement electrodes of the sensor; (**b**) a VNA is connected to the measurement electrode, where the electrode’s output impedance values (*R* and *X*) are shown on the computer screen; and (**c**) the appearance of corn ear.

**Figure 2:**
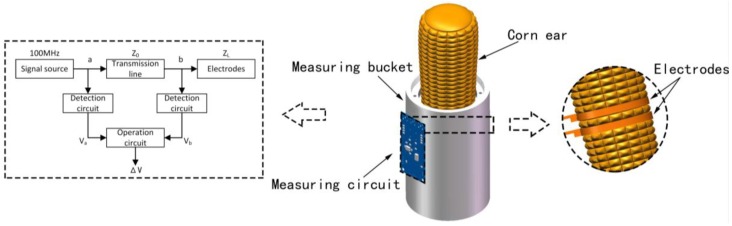
Corn ear moisture measurement sensor structure, including circuit and electrodes.

The principle of our measurement circuit is often used in the field of frequency-domain dielectric sensors [20,21,22,23,24,25], and the measurement apparatus is as shown in Figure 1a. The appearance of the corn ear is as shown in Figure 1c. The corn ear moisture measurement sensor structure is shown in Figure 2; in the structure, a pair of metal ring electrodes (diameter: 50 mm; thickness: 0.2 mm; spacing between the ring electrodes: 0.3 mm; width: 5 mm) is fixed on the measuring bucket. During the moisture measurements, the corn ear was inserted into the measuring bucket to act as the dielectric and produced a capacitance with the electrodes. A high-frequency sinusoidal wave signal was injected into the measurement electrodes through a quarter-wavelength transmission line with a characteristic impedance of *Z*_0_. This leads to signal reflection because of an impedance mismatch between the measurement electrode impedance (*Z_L_*) and the transmission line characteristic impedance (*Z*_0_). Superposition of the reflection signal and the incident signal would form a standing traveling wave signal in the transmission line. Using the transmission line equation $V\left(z\right)=V_{0}^{+}\left(e^{-j\beta z}+\Gamma e^{j\beta z}\right)$ (where *z* is the position on the transmission line, $V^{+}$ is the forward transmission signal amplitude, and β is a transmission coefficient) [26], we can obtain the voltage values at the input and output of the transmission line ($V_{a}=\left|V\left(-\lambda /4\right)\right|=\left|V_{0}\left(1-\Gamma \right)\right|$ and $V_{b}=\left|V\left(0\right)\right|=\left|V_{0}\left(1+\Gamma \right)\right|$), in which the reflection coefficient $\Gamma =\left(Z_{L}-Z_{0}\right)/\left(Z_{L}+Z_{0}\right)$, and λ is the signal wavelength. We then obtain the voltage difference between $V_{a}$ and $V_{b}$ using an arithmetic circuit, where $\Delta V=V_{a}-V_{b}=\left|2V_{0}\Gamma \right|$. Any change in the electrode impedance will cause a change in the reflection coefficient Γ. By measuring ΔV, we can then obtain the change in the load impedance. The electrode impedance is affected by the moisture in the corn, so the corn moisture content can be obtained by measuring the voltage difference at both the beginning and the end of the transmission line. A vector network analyzer (VNA) can measure the electrode impedance in terms of the real value *R* and the imaginary part *X*. We, therefore, studied the electrode’s impedance characteristics using a VNA (E5070B, 300 kHz to 3 GHz, Agilent Technologies, Santa Clara, CA, USA), and the experimental setup is shown in Figure 1b. In addition, based on the transmission line, we have designed a 100 MHz signal source for the high-frequency detection circuit.

### 2.2. Electrode Design

Use of a pair of ring electrodes not only avoids causing physical damage to the corn kernels but also fits well with the geometry of the corn ear. High Frequency Structure Simulator (HFSS release version 13, ANSYS Inc., Canonsburg, PA, USA) [27] software was used in the design of the measurement electrode to study the spatial distribution of electric field, which also contributed to the determination of the measurement electrode’s geometrical parameters. 

**Figure 3:**
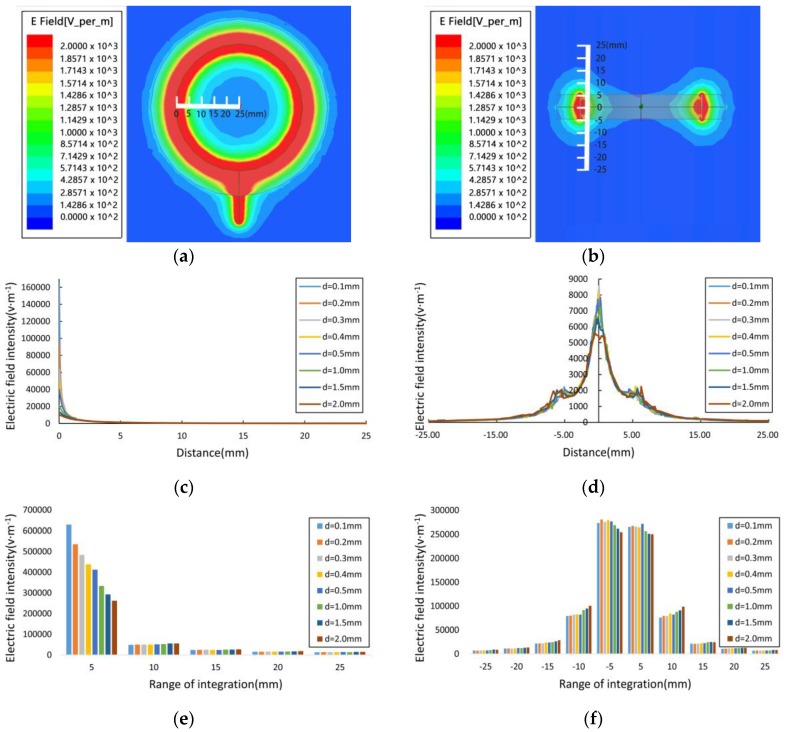
Simulation results for the measurement electrodes. (**a**) Horizontal electric field distribution of the electrode; (**b**) vertical electric field distribution of the electrode; (**c**) electric field intensity distribution curve in the horizontal diameter direction along the electrodes; (**d**) electric field intensity distribution curve in the electrode’s vertical direction; (**e**) electric field distribution histogram in the horizontal diameter direction along the electrode produced by the 5 mm piecewise integral accumulator; and (**f**) electric field distribution histogram in the vertical diameter direction along the electrode produced by the 5 mm piecewise integral accumulator.

Figure 3 shows the simulation results obtained under the condition that the excitation source is set at 100 MHz with the two electrodes spaced at the following distances: *d* = 0.1 mm, 0.2 mm, 0.3 mm, 0.4 mm, 0.5 mm, 1.0 mm, 1.5 mm, and 2.0 mm. Figure 3a,b shows the field distributions between the rings when the two measurement electrodes are spaced at *d* = 0.1 mm. Figure 3a shows the electric field distribution of the electrode in the horizontal direction, while Figure 3b shows the electric field distribution of the electrode in the vertical direction. Figure 3c shows the electric field intensity distribution curve in the horizontal diameter direction of the electrode. Figure 3e shows the electric field histogram distribution that was obtained using a 5 mm piecewise integral accumulator in the horizontal diameter direction of the electrode, and the ruler of the horizontal axis is shown in Figure 3a. From Figure 3c,e, we see that the electric field intensity becomes weak quite sharply in the direction of the electrode’s horizontal diameter, and that the electric field is mainly distributed in the horizontal electric field from the electrodes up to a distance of 5 mm. The electric field intensity is much weaker at distances of more than 10 mm from the electrodes when compared with the intensity at distances of less than 5 mm from the electrodes. As the electrode spacing distance *d* increases, the intensity of the electric field decreases in the horizontal space. From Figure 3e, we see that the electric field intensity near the electrode in the horizontal direction decreases more rapidly than the electric field intensities that were measured far away from the electrode. Therefore, we can enhance the field intensity by simply reducing the electrode spacing or increase the measurement depth of the electrode by increasing the electrode spacing. Figure 3d shows a graph of the electric field intensity distribution along the vertical direction of the electrode, while Figure 3f shows a histogram of the electric field distribution produced by 5 mm piecewise integral accumulation. We, thus, know that the electric field directions are mainly distributed in the −15 mm to +15 mm range in the vertical direction; the experimental setup is shown in Figure 3b.

### 2.3. Experiments on the Effective Scope of the Electrode’s Electric Field

Because a corn ear consists of corn kernels and the corn cob, it is heterogeneous and its structure is obviously layered. It has been found that the moisture contents of the corn kernels and the corn cob are not consistent during the growing period, and are also affected by the storage environment. When we insert a corn ear into the sensor electrodes, the electrode’s electric field will pass through the corn kernels and the corn cob, and because of the effect of the corn ear acting as a dielectric, the electrode’s output impedance will then change. We divided the effects of the electrode’s electric field into horizontal and vertical scope depths and then measured the electrode’s output impedance using a VNA. In the experiments described below, we set the sweep frequency of the E5070B from 300 kHz to 500 MHz, and then acquired the electrode’s output impedance.

#### 2.3.1. Horizontal Effective Scope Depth of the Electrode’s Electric Field

**Figure 4:**
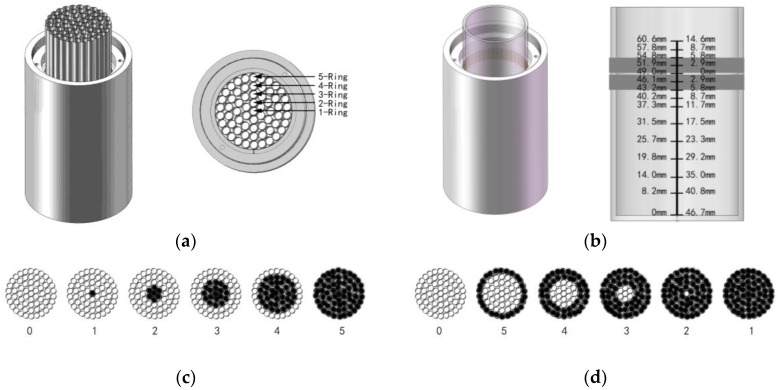
Operation to measure the effective scope of the electrode’s electric field. (**a**) Level influence depth experiment device; (**b**) vertical impact experiment device; (**c**) level influence depth experiments, based on water injection outside introversion; and (**d**) level influence depth experiments, based on outside-in water injection.

To measure the horizontal effective scope depth of the electrode’s electric field, we formed a five-layer cylinder structure using multiple polypropylene (PP) tubes, as shown in Figure 4a, and sealed the bottom of these PP tubes (thickness: 0.1 mm; diameter: 6 mm; dielectric constant: 1.5 at 24 °C). We then placed the cylinder structure into the sensor’s measuring bucket and injected deionized-distilled water (DD water, with conductivity of 2.74 μS·cm^−1^ at 27.6 °C) into the PP tubes. The experiment consists of two parts. The first part involves injection of the DD water into the PP tubes from the first ring to the fifth ring, step by step, as shown in Figure 4c. The second part is the injection of the DD water into the PP tubes from the fifth ring to the first ring, step by step, as shown in Figure 4d. 

#### 2.3.2. Vertical Effective Scope Depth of the Electrode’s Electric Field

To measure the vertical effective scope depth of the electrode’s electric field, we placed a glass beaker (outer diameter: 50 mm; thickness: 2 mm; height: 68 mm; volume: 50 mL) into the sensor’s cylindrical measuring bucket. During the experiment, we added a specific volume of DD water (see Table 1) to the glass beaker. As shown in Figure 4b, we used a ruler to indicate the changes in the DD water’s height and set the middle position of the sensor electrodes as the origin. Its operating steps are shown in Table 1.

### 2.4. Measurement of Sensor Characteristics 

Dielectric theory indicates that the dielectric properties of the dielectric material under the varying electric field are affected by the interfacial polarization, the dipolar polarization, the ionic polarization, and the electronic polarization. Since water molecules are polar molecules, the dielectric properties of the water are mainly dependent on the dipolar polarization; the dipolar polarization is closely related to the temperature and, thus, the temperature is an influential factor for the moisture content detectors, which were designed on the basis of the theory of dielectrics. Additionally, the existence of salinity in the corn kernels can generate ionic polarization and, thus, the conductivity is another influential factor. To determine the effects of the above factors on the sensor, the following related experiments were conducted.

#### 2.4.1. Effects of Temperature Variation

In the experiments to determine the effects of temperature, a PP measuring cup with wall thickness of 0.1 mm and permittivity of 1.5 at 24 °C was placed into the sensor. The experiments included gradually reducing high temperatures and gradually increasing low temperatures, and a Fluke 52 II dual input digital thermometer (Fluke Corporation, Phoenix, AZ, USA) was used to acquire the temperature data. In the experiment based on gradual reduction from a high temperature, a specified amount of DD water at 90 °C was poured into the measuring cup, and the decreasing temperature was monitored via the thermometer; for every approximately 10 °C reduction in temperature, the sensor’s output impedance was measured using the VNA or the differential output voltage was obtained by the detection circuit. In the experiment based on a gradual increase in temperature to reach a high temperature, a specific amount of an ice water mixture made from DD water was poured into the measuring cup; after the ice in the ice water mixture melted completely, the related data were recorded as described above for every increase of approximately 5 °C.

#### 2.4.2. Effects of Conductivity Variation

In the experiments to determine the effects of conductivity variation, a specified amount of DD water was poured into the measuring cup in the sensor, as described above. 0.5 g of KCl was divided into 10 equal parts, and these portions were gradually added to the DD water. For each addition, after the KCl had dissolved completely, the conductivity of the KCl solution was measured using a CDH222 conductivity/TDS (Total Dissolved Solids) meter and the sensor’s output impedance was measured using the VNA.

### 2.5. Experiments for Corn Sample Measurement

To verify the effectiveness of the sensor’s measurements, we measured some ripe corn samples using the corn ear moisture sensor. The measured corn samples include two varieties of corn, JH1303 and Jindao111, which were provided by the Beijing Kings Nower Seed Science and Technology Co. Ltd., Beijing, China). The ripe corn samples were taken from the breeding base in Hainan, and the initial moisture content of each corn ear was approximately 35%. First, we selected 30 samples of each type of ripe corn ear, where the ear diameter of the corn is 48–53 mm. Then, we conducted the experiments as described below:

(1) Five to 10 selected samples were measured using the corn ear moisture sensor and the output impedance of the sensor’s electrodes was acquired using the VNA at the same time;
(2) The test corn samples were threshed, and the absolute moisture value was obtained via the oven-drying method at 130 °C for 24 h. The reference moisture value is calculated as follows:
  $$corn\;kernel\;moisture=\frac{wet\;kernels\;mass-dry\;kernels\;mass}{wet\;kernels\;mass}\times 100\text{\%}$$
(3) The moisture in the remaining selected corn samples was allowed to dry naturally for one to two days, and Steps (1) and (2) were repeated.

**Figure 5:**
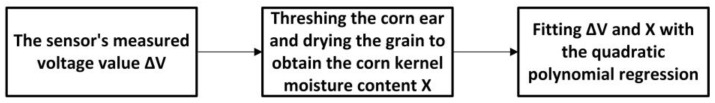
Rapid nondestructive process for measurement of the moisture content of the kernels of a corn ear.

The rapid nondestructive process for measurement of the moisture content in the kernels of a corn ear is shown in Figure 5.

## 3. Results

### 3.1. Experiments to Determine Effective Scope of the Electrode’s Electric Field

**Figure 6:**
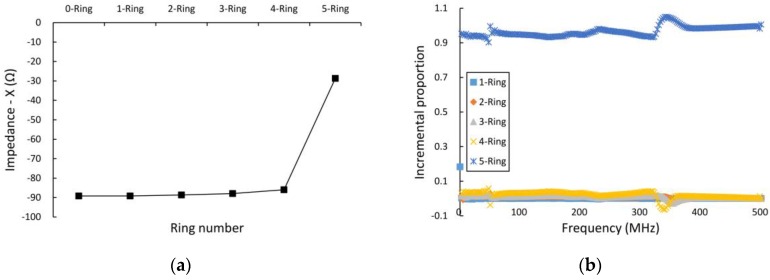

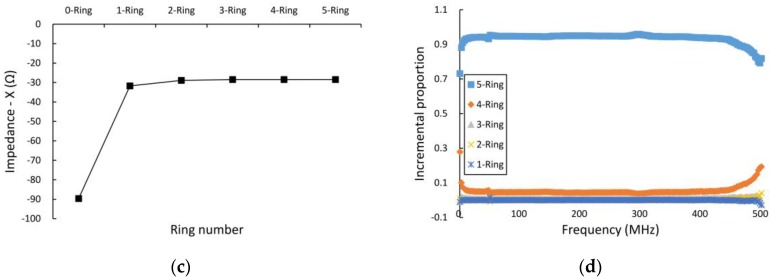
Results of horizontal effective depth measurement of the electrode’s electric field. (**a**,**b**) show the imaginary part of the electrode’s output impedance versus injected DD water in the PP tubes from the first ring to the fifth ring; (**c**,**d**) show the imaginary part of the electrode’s output impedance versus DD water injection into the PP tubes from the fifth ring to the first ring.

The overall results of measurement of the horizontal effective scope of the sensor electrode ring’s electric field are shown in Figure 6. As shown in Figure 6a,b, the imaginary part of the electrode’s output impedance varied, and it was also changed by injecting DD water into the PP tubes from the first ring to the fifth ring, with the sweep frequency ranging from 300 kHz to 500 MHz. Figure 6c,d shows that the imaginary part of the electrode’s output impedance varied when DD water was injected into the PP tubes from the first ring to the fifth ring, and the frequency was swept from 300 kHz to 500 MHz. Figure 6a,c indicates that the fifth water ring (which is located closest to the sensor electrodes) has a dominant effect on the variation in the electrode’s output impedance. We defined a variable $\eta =\left(X_{n}-X_{n-1}\right)/X_{\mathrm{sum}}$ to represent the percentage of a specified layer’s effect on the imaginary part of the electrode output impedance. As shown in Figure 6b,d, the η of the fifth water ring (located closest to the sensor electrodes) is approximately 0.98, while the total η for all other water rings is just 0.02; the total η of all water rings shows a weak correlation with the sweep frequency over the range from 300 kHz to 500 MHz. The horizontal effective scope of the electrode’s electric field is, thus, nearly 6 mm (i.e., the diameter of a single PP tube). The length of a typical corn kernel of the corn samples under test is approximately 6 mm, so this scope mainly affects the corn kernels in the corn ear. The corn kernel length is in the 8–15 mm range, i.e., the thickness of the kernel layer in Figure 1c. The electrode, thus, mainly measures the water content in the layer of kernels.

**Figure 7:**
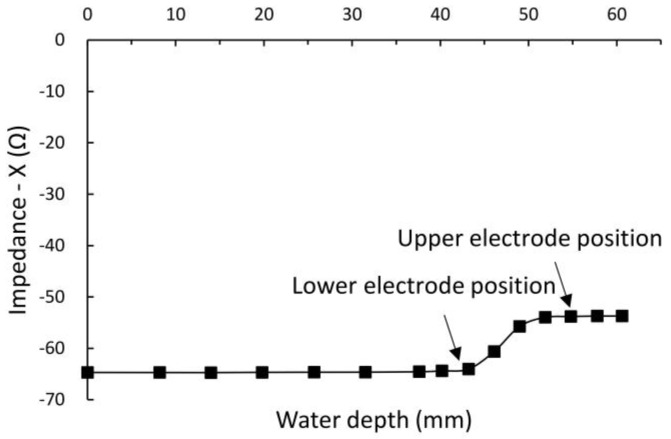
Results of measurement of the vertical effective depth of the electrode’s electric field.

As shown in Figure 7, the vertical effective depth of the electrode’s electric field is strongly related to the positions and widths of the sensor electrodes. When we injected approximately 70–80 mL of DD water, the imaginary part of the output impedance of the sensor electrodes showed obvious variation. Simultaneously, the water surface increased from 43.2 mm to 51.9 mm (43.2 mm is the electrode’s lower boundary, and 54.8 mm is its upper boundary). Therefore, the vertical effective depth of the electrode’s electric field is nearly equal to the width of the sensor electrodes.

The experiments showed that the electric field distributions in the horizontal and vertical directions of the electrode are concentrated in a very small space, which is consistent with the electrode electric field simulation results above.

### 3.2. Measurement of Sensor Characteristics 

#### 3.2.1. Effects of Temperature Variation

**Figure 8:**
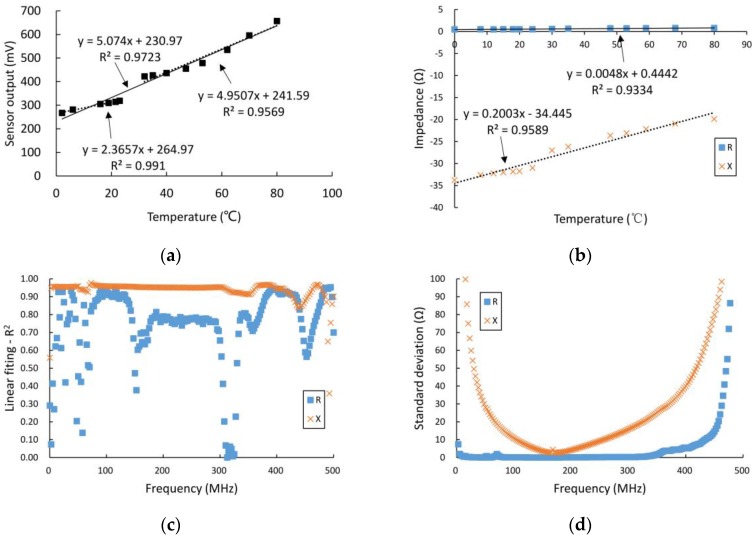
Results of experiments on temperature variation effects. (**a**) The relationship between temperature and differential output voltage from the detection circuit at a frequency of 100 MHz; (**b**) The relationship between temperature and output impedance from the VNA at a frequency of 100 MHz; (**c**) coefficients of determination *R*^2^ in the frequency range from 300 kHz to 300 MHz; and (**d**) standard deviations in the frequency range from 300 kHz to300 MHz.

The results of the measurements, in which the DD water was measured for a series of temperatures in the range from 0 °C to 80 °C using the self-designed sensor circuit and the network analyzer, are shown in Figure 8a,b. As shown in the figures, when measured at the operating frequency of 100 MHz, the differential output voltage from the detection circuit and the real and imaginary parts of the output impedance from the VNA are all highly linearly correlated (coefficients of determination *R*^2^ are 0.9723, 0.9334, and 0.9589, respectively) with the measured temperatures. The slope of the linear relationship between the real part of the output impedance and the temperature is 0.0048, while that of the imaginary part is 0.2003; the temperature variation, thus, mainly affects the imaginary part of the output impedance, but it also has a relatively small effect on the real part. As shown in Figure 8c, in the frequency range from 300 kHz to 500 MHz, the imaginary part of the output impedance always has a much better linear relationship with the temperature values than the real part. Figure 8d shows the values of the standard deviation between the sensor’s output impedance and the temperature in the measured frequency range, as described above. In the frequency range from 300 kHz to 300 MHz, the values of the standard deviation for the real part of the output impedance are less than 1 Ω, indicating that the effect of the temperature is very little, but when the frequency increases beyond 300 MHz, the standard deviation values increase rapidly and, therefore, the effect of the temperature also increases rapidly. The effects of the temperature on the imaginary part of the output impedance initially decrease before increasing, and the imaginary part reaches a minimum at a frequency of approximately 160 MHz.

#### 3.2.2. Effects of Conductivity Variation

**Figure 9:**
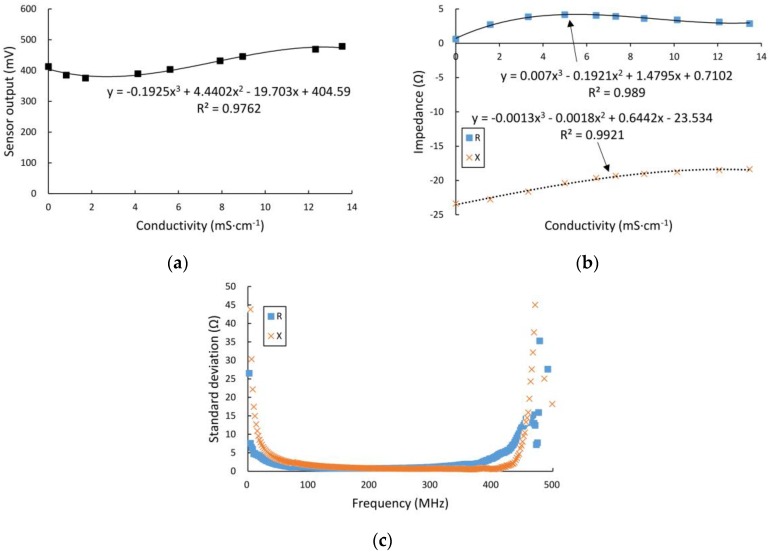
Results of experiments on conductivity variation effects. (**a**) The relationship between conductivity and the differential output voltage from detection circuit at frequency of 100 MHz; (**b**) the relationship between conductivity and output impedance from the VNA at a frequency of 100 MHz; and (**c**) standard deviations at various frequencies ranging from 300 kHz to 300 MHz.

The results of the experiments using the self-designed sensor circuit, in which the KCl solution conductivities had a series of values in the range from 0.001 to 13.55 mS·cm^−1^, are shown in Figure 9a. Meanwhile, experiments were also performed using the network analyzer, and the conductivities of the KCl solutions are shown to range from 0.001 to 13.46 mS·cm^−1^ in Figure 9b. As shown in these figures, at the operating frequency of 100 MHz, the differential output voltage from the high-frequency detection circuit and the real and imaginary parts of the output impedance from the VNA are not linearly correlated with the measured conductivities in this case, but all three relationships are cubic polynomials. Figure 9c shows the values of the standard deviation between the sensor’s output impedance and the conductivity at the different frequencies in the range from 300 kHz to 500 MHz. These results indicate that, as the frequency increases, the effects of conductivity variation on the real and imaginary parts of the output impedance both initially decrease and then increase, and reach their own minima at frequencies of approximately 200 MHz and 300 MHz, respectively.

### 3.3. Experimental Measurements of Corn Samples

**Figure 10:**
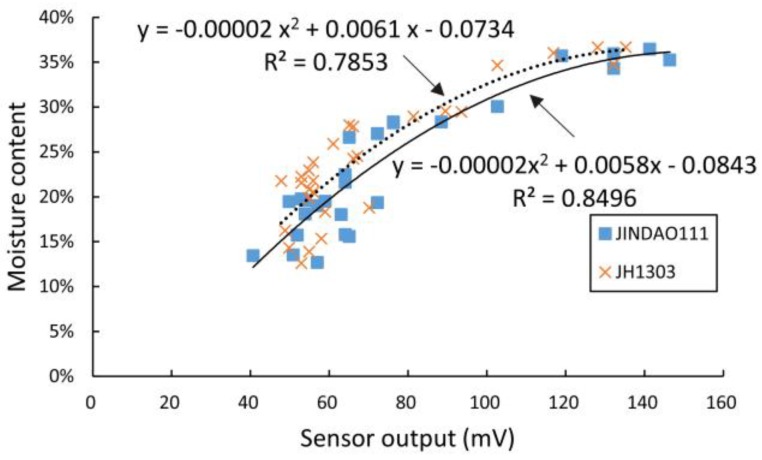
Output voltage of the sensor’s detector circuit versus absolute moisture of the corn under test.

**Figure 11:**
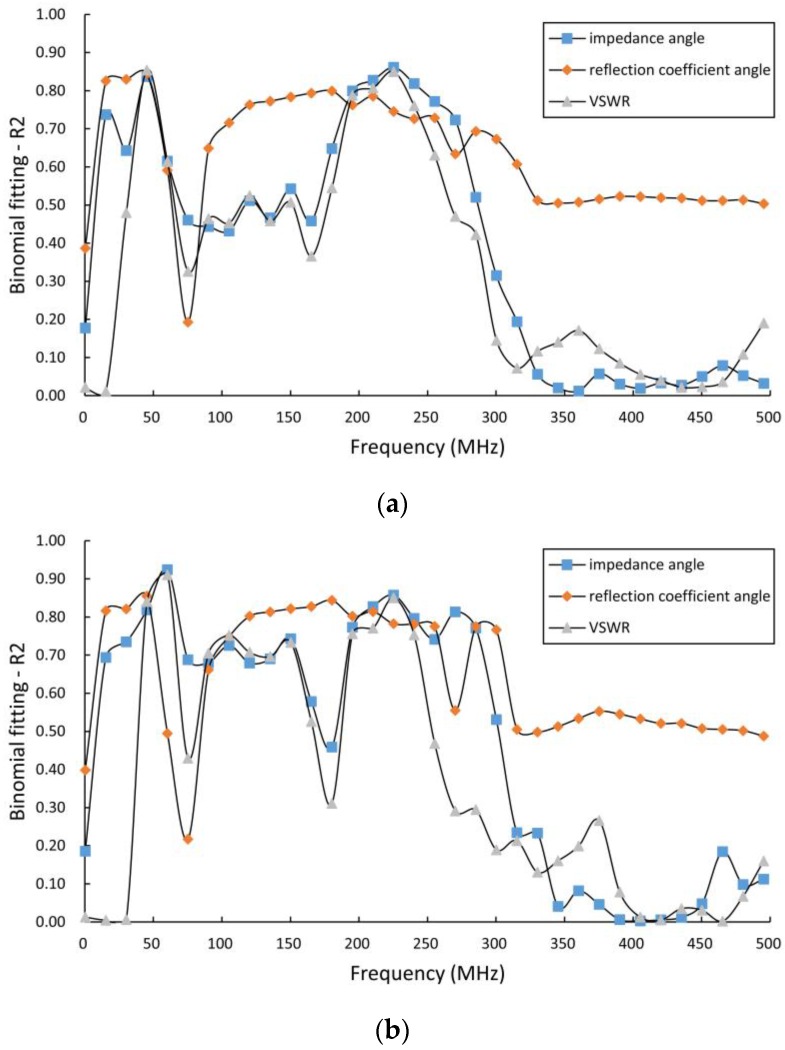
*R*^2^ of the quadratic polynomial fitting over the sweep frequency range. (**a**) Corn: JINDAO111; and (**b**) corn: JH1303.

In Figure 10, we have plotted the output voltage of the sensor’s detector circuit relative to the absolute moisture of the corn samples under test. As the figure shows, the relationship between the output voltage and the absolute moisture content of the corn kernel samples (JINDAO111 and JH1303, Beijing Kings Nower Seed Science & Technology Co. Ltd., Beijing, China) is consistent with quadratic polynomial fitting, and the coefficients of determination *R*^2^ of the two samples were 0.7853 and 0.8496, respectively. We then calculated the voltage standing wave ratio (VSWR), the phase angle of the output impedance, and the reflection coefficient from the data that were acquired by the VNA in the sweep frequency range from 300 kHz to 500 MHz. Finally, we fitted the VSWR, the phase angle described above and the absolute moisture of the corn samples under test using a quadratic polynomial, and plotted the coefficient of determination *R*^2^ over the sweep frequency range from 300 kHz to 500 MHz. As shown in Figure 11, the maximum coefficient of determination *R*^2^ occurs in the frequency range from 45 to 60 MHz and at a frequency close to 225 MHz. 

## 4. Discussion

For in situ measurements of corn ear moisture, the measured value should mainly carry information about the moisture content contribution of the corn kernels. Therefore, the structural parameters and profile of the sensor must be carefully designed to eliminate or minimize the effects of the cob on the measurements. First, we used the HFSS simulation software to model the electric field distributions for a series of structure setting parameters of the sensor, which allowed the electric field to be spatially distributed in an appropriately limited range close to the electrodes. As shown in Figure 3, the electric field strength in the radial direction attenuates dramatically when the position is more than 10 mm from the electrode when compared with that measured at a distance of 5 mm. Fortunately, the length of the corn kernels ranges from 8 mm to 15 mm and is, thus, in the sensitive range that will allow the detectors to measure the moisture content of the corn kernels. Next, we designed an experiment using the sensor to verify the simulation results, and the results indicated that the corn cob has little effect on measurement of the moisture content. The measurement results showed that, along the direction of the electrode ring radius, the effect of the dielectric medium on measurement of electric field at a distance of more than 10 mm from the electrode was less than 2.04%; see Figure 4c,d and Figure 6a,c. Both results confirmed that the designed sensor was suitable for measurement of the moisture content of corn kernels, and that the corn cob had very little effect on the measurements. There were few reports of in situ non-threshed measurement of corn kernels moisture. Reid et al. [14] used a wood moisture meter model MT808 which was modified with two steel pins, by penetrating the husk and kernels to measure kernel moisture content. Compared to our method, the previous work indicated that cobs influenced in suit moisture measurements of corn kernels, when kernels moisture was between 20% and 40%.

Additionally, the results indicated that the standard deviation of the real part of the impedance is less than 1 Ω in the frequency range from 300 kHz to 300 MHz when the temperature varied from 0 to 80 °C. For this reason, the appropriate frequency was set at 100 MHz.

Figure 9 shows that while there was little change in the output signal when the electrical conductivity of the ionic solution is in a wider range from 0.001 to 13.46 mS·cm^−1^, the signal variation is less than 1% if the change in the electrical conductivity is less than 1 mS·cm^−1^. The electrical conductivity of corn kernels is well known to decrease gradually with slow water loss under natural conditions; in our experiments, the electrical conductivity of the corn kernels is 0.04 mS·cm^−1^ at 11.4%, but 0.496 mS·cm^−1^ at 36.5%, and this suggested that the signal change caused by the variation of the electrical conductivity is less than 1% at 100 MHz.

As shown in Figure 1c, the profile of a corn ear is like the frustum of a cone, which makes it easy to couple the designed sensor to a corn ear if its diameter is in the appropriate range. Here, the electrode diameter in the sensor is 50 mm. Our results indicated that there is an adaptable coupling when the electrode diameter is within the range from the diameter near the tip (45 ± 3 cm) to the diameter of the butt end (52 ± 1 cm). For in situ measurement of the moisture content of a corn ear, the corn ear was inserted slowly into the ring-shaped electrode of the sensor. The experimental results show that as long as the electrode ring diameter is in the range between the diameters at the butt end and that at the tip of the corn ear samples, the electrode ring can be coupled tightly with the corn ear in some positions. In this paper, we place the corn ear in the sensor electrode ring with the corn butt end at the top and the tip at the bottom, relying on its own gravity, and the contact with the electrode ring will not cause crush injuries to the kernels in the corn ear. In future work, a series of electrodes of different sizes will be developed to enable a wider application of the method.

In fact, there are many different kinds of corn kernel shapes, e.g., flint corn, dent corn, and semi-dent corn, and the gap spaces among the corn kernels are different for each type of corn that may affect the measurements. However, for the semi-dent corn, the JINDAO111 and JH1303 samples showed no significant differences from each other in the different gap spaces among the corn kernels. In general, it is worth investigating issues with regard to the effects of the gap space among the corn kernels on in situ measurement of the corn ear moisture content.

In this study, to verify our proposed in situ measurement method and our sensor design, we used two different types of corn ears (JINDAO111 and JH1303) with moisture contents in the range from 12.59% to 36.5%. While there are many available maize varieties with different types of corn kernel shapes, our operation is able to reduce the complexity at this stage, and enable research from simple to sophisticated levels. In future studies, we intend to measure more corn varieties using the developed method. 

Additionally, we focused on development of the novel method and provided a preliminary prototype sensor for in situ measurement of the moisture of a corn ear, but there is still room for improvement in changing the sensor into a ring structure with two electrodes such that the volume is reduced, making it easy to carry the sensor to the required scene from a practical perspective. At present, four methods, i.e., DC conductance, radio-frequency impedance, microwave cavity, and nuclear magnetic resonance techniques, are able to measure the moisture content of the threshed corn kernels, even a single kernel [7]. Funk et al. [7] investigated the variation in moisture readings of threshed corn kernels due to influencing factors, e.g., density, and techniques were proposed for their corrections with measurements at 149 MHz. Thus, the density correction will be considered in our design. Furthermore, we also will intend to improve performance by the design of an ultralow-power portable apparatus that relies on common and popular techniques for embedded system design.

## 5. Conclusions 

To meet measurement requirements for maize breeding, we developed a noninvasive in situ measurement method and a reliable prototype sensor to determine corn kernel moisture content. The moisture content is sensed based on correlations with the dielectric properties of the corn kernels. In this work, we investigated the effects of the sensor profile on the electric field distribution and the moisture readings through both simulations and experiments. To reduce the influence of the electrical conductivity, we used a measurement frequency of 100 MHz to detect the moisture content. The experimental results proved that the proposed method has a wide moisture measurement range from 12.59% to 36.5% with a rapid sensing time of less than 1 s. In future studies, we intend to use a series of electrodes of different sizes to enable wider application requirements.

## Figures and Tables

**Table 1:** Volume of injected DD during points where the vertical electric field affects the measurements *.

No.	Injecting Volume (mL)	Total Volume (mL)	Water Level (mm)	Relative Height (mm)
1	10	10	8.2	−40.8
2	10	20	14.0	−35.0
3	10	30	19.8	−29.2
4	10	40	25.7	−23.3
5	10	50	31.5	−17.5
6	10	60	37.3	−11.7
7	5	65	40.2	−8.7
8	5	70	43.2	−5.8
9	5	75	46.1	−2.9
10	5	80	49.0	0
11	5	85	51.9	2.9
12	5	90	54.8	5.8
13	5	95	57.8	8.7
14	5	100	60.6	14.6

* Properties of DD water: conductivity of 2.74 μS·cm^−1^ at 27.6 °C.
